# Editorial: COVID-19 and Behavioral Sciences

**DOI:** 10.3389/fpubh.2021.830797

**Published:** 2022-01-14

**Authors:** Tracy Jackson, Liz Steed, Rebecca Pedruzzi, Kebede Beyene, Amy Hai Yan Chan

**Affiliations:** ^1^Usher Institute, The University of Edinburgh, Edinburgh, United Kingdom; ^2^Institute of Population Health Sciences, Queen Mary University of London, London, United Kingdom; ^3^Telethon Kids Institute, University of Western Australia, Perth, WA, Australia; ^4^School of Pharmacy, Faculty of Medical and Health Sciences, The University of Auckland, Auckland, New Zealand

**Keywords:** public health, healthcare, health protection, risk behavior, behavioral sciences, COVID-19, psychology

In March 2020, COVID-19 was declared a pandemic threatening the well-being of people, health systems, and global economies if not managed appropriately (1). Even with the development of effective vaccines and treatments for COVID-19, measures to manage the spread of infection depend upon the behavior of individuals and their influence on complex public health systems. The application of behavioral sciences to promote adherence to public health and health promotion measures toward protection from COVID-19, such as hand hygiene, mask wearing or social distancing, have been integral to containing and limiting the spread of COVID-19 within our societies. Adherence to the health protection measures is crucial for determining the outcome of the COVID-19 pandemic, and the impact on society.

The outbreak of COVID-19 during a time when technology, digital engagement, and social media are widely used, has given rise to an “*infodemic*”—a portmanteau of “*information*” and “*epidemic*”—the abundance of information (including false and unsubstantiated evidence such as misinformation and disinformation) during a disease outbreak (2). Balancing the optimal provision of accurate information, that is constantly changing as knowledge and understanding of the novel COVID-19 grows, with clearly defined public health messages required to modify individual behavior is necessary, but challenging. The infodemic is recognized to be a key contributor to the behavioral response to COVID-19, and may have fuelled vaccine hesitancy, due to misinformation and disinformation spread through media, social media and online sources (3).

The aim of this Research Topic “COVID-19 and Behavioral Sciences” was to identify and explore the factors that influence the behaviors of individuals during the COVID-19 pandemic- in particular why some individuals undertake at-risk behaviors such as lack of hand hygiene. The Topic had 60 submitted manuscripts with a final 34 articles accepted, exploring the application of behavioral sciences to the management of the pandemic, and the impact of behavior on the spread or containment of the COVID-19 pandemic. This is a global collection containing 16 articles from Europe; 15 articles from Asia; 1 from North America, 1 from Africa, and 1 multinational study with participants from 7 Latin American countries.

The Research Topic had overall six overarching themes:

1) Risk communication and public health messaging

Clear communication in public health messages is required for understanding and adherence. Stroom et al. discussed the public health policy recommendation to “*avoid crowded places*” in the Netherlands viewing it as subjective, open to individual interpretation and ultimately potentially counterproductive as when more people ventured out this allowed others to legitimize their violation of restricted movement guidelines. The interplay between trust in science and news media coverage of COVID-19 was explored by Neureiter et al. Trust in science affected how exaggerated the media were perceived, with the less the media was perceived to be exaggerated, the more likely individuals were to participate in health protective behaviors. In the United Arab Emirates, websites (health information websites), social media, government communications, and family and friends were the most frequently accessed sources of information on COVID-19, as identified by Figueiras et al., however family physicians, health care professionals, and government communications were perceived as the most trustworthy information sources. Information and misinformation on the emergence of COVID-19 has led to stigmatization of certain social groups. Chen X. et al. explore the negative impact of this in an online survey of 313 participants in China. In this study, social stigmatization was shown to have a significant negative impact on emotions, and the stronger the perceived level of stigma by the stigmatized groups, the more anger, anxiety, and grief they will have. Interestingly, this study also demonstrated that negative emotions among inflicted groups can promote social altruistic tendency among group members. The authors advised caution against baseless accusations and targeting specific social groups and emphasized the importance of combating stigma in an effort to recover from this pandemic.

2) Public education and health literacy

The COVID-19 pandemic has increased public awareness of the concept of public health education. Shen et al. found that only 22.1% of Chinese citizens in their study were aware of “public health” prior to the pandemic. However, by February 2021, their cross-sectional study found that 74.5% were supportive of public health education and heavily influenced by economic status, personal perception, and understanding. Reductions in health literacy in 2020 reported by Yang et al. strengthened the call for tailored interventions according to health literacy in different age groups and different socioeconomic backgrounds, to minimize the equality gap between rural and urban health in China. Bukuluki and Kisaakye found that more than three quarters of participants of the study in the urban Greater Kampala Metropolitan area believed in the efficacy of facemasks and wore facemasks as a preventative health measure. There was no significant difference between facemask wearing in indoor or outdoor public spaces, but this was not universal and more information and education is required for rural areas.

3) Community engagement

However, behavior change is not always possible for all members of society, especially those communities who are at higher risk of serious health outcomes due to inequities influencing social determinants of health. Lauwerier et al. highlight the need to effectively engage communities in strategy development to ensure the relevance and acceptability of prevention approaches. In Oman, a range of three community participation methods were undertaken to address rising COVID-19 cases: community organizations within cities and villages; district health committees with collaboration at state level; community volunteers Al Siyabi et al. Intimate knowledge of the logistics of communities is held by their members, and community participation empowers them to identify risks and needs and mobilize individual members to collaborate for positive impact. Updates to the online hand washing intervention, Germ Defense, are described by Morton et al., including the addition of a “Reducing Illnesses” component. The added component was developed with significant involvement by patient, public and clinical stakeholders, delivering an intervention that was relevant and engaging to users during the COVID-19 pandemic.

4) Psychological impact of COVID-19

The psychological impact of COVID-19 must not be under-estimated, Caycho-Rodríguez et al. found that in 4,881 participants from seven Latin American countries, depressive symptoms, anxiety and fear of COVID-19 were commonly identified with a quarter of participants reporting generalized anxiety disorder symptoms and a major depressive episode. It is evident that forced confinement and social distancing measurements during the COVID-19 pandemic have negatively impacted the mental health of individuals'. Peterson's et al. study in the United States reported findings of increased depressive and anxiety symptoms with a decrease in mood over time with social isolation and social distancing practices. The psychological impact was explored further by Fenollar-Cortés et al. in 164 participants where there was a significant gender difference at the start of forced confinement in Spain, with women having higher scores for depression, anxiety, stress, and intrusive/avoidance symptoms. By the end of forced confinement, women had managed to significantly improve their scores across most of the psychological measures. These gender differences were also evident in the United Arab Emirates– Al Miskry et al. concluded that more females than males experienced a risk of psychological problems and were more likely to use avoidance and emotion focused techniques than men to cope with the lockdown. Interestingly, increased social interaction in the first stages of forced confinement negatively impacted well-being and increased stress in individuals in Vienna, as reported by Kim and Florack. More social communication resulted in the social amplification of information about COVID-19 which was associated with higher incidences of panic buying and a decreased trust in society. However, social support was positively correlated with post-traumatic growth in discharged COVID-19 patients (Yan et al.).

5) Coping strategies and the COVID-19 pandemic

The importance of coping strategies for the protection of individuals' physical and mental health during the COVID-19 pandemic were recognized across several studies which drew on behavior change theories to help frame their work. Sousa et al. drew on self-regulation theory (4) and found self-regulation and healthy habits to be a good indicator of the adoption of a healthier lifestyle and improved mental health during the pandemic in Portugal. However, self-regulation became more difficult as the duration of restricted movement extended. The study by González-Castro et al. drew on the Health Belief Model (5) and suggests individual's perceived severity and vulnerability to COVID-19 are related to the utilization of protective health behaviors, dependent on exposure to COVID-19 and perceived self-efficacy moderators. For those not directly exposed to COVID-19, recognition of individual ability to prepare for situations and undertake instrumental actions, such as carrying hand sanitizer and face masks, facilitated adherence to protective behaviors. Not all coping strategies undertaken by individuals are healthy, for example, tobacco use. In a study of 700 people in China, Feng et al. investigated smoking cessation intention in the context of the COVID-19 pandemic. The authors recognized that addressing these behaviors alongside the pandemic has been challenging, in part due to the contradictory messages coming from the media concerning the potential preventative nature of cigarette smoking on COVID-19. Integrating two psychological models in their questionnaire, based around the Integrative model derived from the Theory of Planned behavior (6). Feng et al. identified that positively-valenced messages of the impact of smoking on the prevention and treatment of COVID-19 were significant in predicting the intention to quit smoking during the pandemic, with the positively-valenced messages indirectly predicting support for tobacco control measures. In contrast, perceived susceptibility, barriers and subjective norms had no impact.

6) Adherence to public health preventive recommendations

Most of the Research Topic papers explored factors that influence adherence to public health recommendations. In a three round nationwide cross-sectional panel survey of over 1,000 adults between July and November 2020, Rodríguez Blázquez et al. reported that there were high levels of knowledge of COVID-19 amongst the general population in Spain, and most respondents supported face masks and the night curfew. However, risk perception and self-efficacy were low; the perceived probability of contracting COVID-19 remained constant but the perceived severity of getting infected decreased over time. In addition, 41–49% of respondents believed they would be unlikely to contract COVID-19 when meeting with family and friends. In India, the wearing of face masks was the most reported preventative behavior in Lahiri's et al. study of 2,646 adults, followed by washing hands with soap and water. However, only a small proportion of the study participants (<10%) were regularly practicing all COVID-19 preventive behaviors investigated. Those with higher perceived severity and vulnerability to COVID-19 were more likely to participate in protective health behaviors. The authors noted the importance of promoting synergistic behavioral practices through appropriate risk communication strategies. Šurina et al. report similar findings in their study of 2,608 online survey participants in Latvia where those with higher COVID-19 threat appraisal experienced higher levels of fear of COVID-19 and were more adherent to COVID-19 preventative behaviors. On the other hand, COVID-19 conspiracy beliefs were negatively correlated with COVID-19 threat appraisal and trust in COVID-19 information sources, but were not a significant predictor of COVID-19 preventive behaviors.

Cerami et al. discussed how risk aversion increased with age with younger people believing they were less vulnerable to the serious outcomes of COVID-19. Those with a more general tolerance of risk tended to believe more in external factors controlling health status rather than internal. Those with higher risk aversion believed their behavior could protect them from contracting COVID-19 and were more likely to participate in health preventative measures. Park et al. investigated the interaction between perceived severity of COVID-19 and adherence to preventative behavior between the first and second waves of the pandemic in their study of 1,144 adults in South Korea. While perceived severity of COVID-19 increased by the second wave, the perceived importance of social distancing did not increase and shifted from being a voluntary to a compulsory behavior. Individuals' adherence to health protective measures remained high throughout the pandemic, however Han et al. found adherence declined as the pandemic progressed.

For university students in Norway, a range of behavior change techniques, including email updates from their institution, visual reminders and provision of antibacterial dispensers, were well-received, as reported by Vande Velde et al. Student's trust in their institution had the strongest effect on the effectiveness of email updates, whereas existing attitudes toward infection control behaviors were more impactful than the visual reminders and novel opportunities for health promotion. Services that supported individuals to adhere to public health guidelines were popular, reflected by the reported rise in online food services by Sakai et al. These services enabled individuals to remain at home rather than venture out for essential sustenance. However, the increase in online services to order food was not sustained and participants' intentions for going out activities in the future when restrictions eased were higher compared to pre-pandemic levels of going out activities.

Neto et al. explored the role of illness perceptions related to understanding COVID-19 in their pan-European sample of 7,032 participants. The authors reported a change in illness perceptions over time with females increasing their perceived sense of personal and treatment control. In Tagini's et al. study of 964 Italian adults, high levels of anxiety, an anxious attachment style, and an external locus of control predicted higher perceived risk. The higher the perceived risk, the more likely protective health behaviors were adopted. In Chen S. et al. study of 896 individuals in 3 Chinese cities, Wuhan, Hangzhou, and Jinan, internal (optimistic bias) and external reference points (social norms) determined individuals' adoption and adherence to health protective behaviors. Cultural attributes, including a high level of egalitarianism and hierarchy and a low level of individualism and fatalism, were identified by Bi et al. as being significantly associated with protective health behaviors in their study of 17,651 adults in mainland China. Xu et al. examined the effects of festivities on health promoting behaviors during Chinese New Year 2020 and Summer 2020, and found participants were more likely to have increased adherence to infection-prevention behaviors and reported fewer fear responses and less attention paid to the COVID-19 pandemic. The participants perceived a high likelihood of being infected and community risk but balanced this with an increase in health protection behaviors and a reduced psychological response.

Vaccines have been viewed as a roadmap out of restricted movement mandates, but misinformation surrounding the speed of their development and the potential long term impact on individual health has raised questions among the publics. Wolff reported intentions to receive a COVID-19 vaccine were predicted by positive attitudes toward COVID-19 vaccines, subjective norms in favor of vaccination in individuals' families, and perceived behavioral control. In Rosman's et al. study of 314 university students in Germany, those who believed in the necessity of expertise and authority within medicine, were more likely to report vaccination intentions. However, prosocial values did not elicit the same intentions.

## Summary

Overall, the 34 included papers in this special edition are an exploration of behavioral sciences in health protection during the COVID-19 pandemic. The rollout of effective COVID-19 vaccines has not removed the need for health protections measures–rather vaccines are an essential component of a holistic public health response to protecting the health of people, health systems, and global economies. Adherence to public health recommendations, guidelines, and restricted movement mandates are influenced by social norms, self-efficacy, and perception of risk. As seen from our Special Topic article collection, behavioral sciences are integral to developing effective public health interventions that motivate individuals to partake in health protective behaviors. Involving patients, the public and clinical stakeholders ensure that the measures are accessible, sustainable, beneficial, and relevant to those they intend to impact.

## Author Contributions

All authors conceptualized and contributed to the article and approved the submitted version.

## Funding

TJ was supported by Asthma UK as part of the Asthma UK Center for Applied Research (Grant Nos. AUK-AC-2012-01 and AUK-AC-2018-01) and RESPIRE, outside the current work. RESPIRE was funded by the National Institute for Health Research (NIHR) (16/136/109) using UK aid from the UK Government to support global health research. KB reports grants from Health Research Council of New Zealand, Amgen, A+ Charitable Trust (Auckland District Health Board), Universitas 21, New Zealand Pharmacy Education and Research Foundation, the University of Auckland, and Addis Ababa University outside this work. AC is a fellow of, and supported by Asthma UK as part of the Asthma UK Center for Applied Research (Grant Nos. AUK-AC-2012-01 and AUK-AC-2018-01). AC reports grants from Innovate UK, A+ Charitable Trust (Auckland District Health Board), Maurice and Phyllis Paykel Trust, Universitas 21, New Zealand Pharmacy Education Research Fund, Auckland Academic Health Alliance, Asthma UK, Health Research Council, Chorus Ltd, Oakley Mental Health Foundation and the University of Auckland; reports consultancy fees from Janssen-Cilag and UCL-Business spin-out company Spoonful of Sugar Ltd; and is the recipient of the Robert Irwin Postdoctoral Fellowship, outside the submitted work.

## Author Disclaimer

The views expressed in this publication are those of the author(s) and not necessarily those of the NIHR or the UK Department of Health and Social Care nor views of any of other funding bodies.

## Conflict of Interest

The authors declare that the research was conducted in the absence of any commercial or financial relationships that could be construed as a potential conflict of interest.

## Publisher's Note

All claims expressed in this article are solely those of the authors and do not necessarily represent those of their affiliated organizations, or those of the publisher, the editors and the reviewers. Any product that may be evaluated in this article, or claim that may be made by its manufacturer, is not guaranteed or endorsed by the publisher.
